# Implementing artificial intelligence in breast cancer screening: Women’s preferences

**DOI:** 10.1002/cncr.35859

**Published:** 2025-04-22

**Authors:** Alison Pearce, Stacy Carter, Helen ML Frazer, Nehmat Houssami, Mary Macheras‐Magias, Genevieve Webb, M. Luke Marinovich

**Affiliations:** ^1^ The Daffodil Centre The University of Sydney A Joint Venture With Cancer Council New South Wales Sydney New South Wales Australia; ^2^ Sydney School of Public Health The University of Sydney Sydney New South Wales Australia; ^3^ Australian Centre for Health Engagement, Evidence and Values School of Health and Society University of Wollongong Wollongong New South Wales Australia; ^4^ St Vincent’s Hospital Melbourne Fitzroy Victoria Australia; ^5^ BreastScreen Victoria Carlton Victoria Australia; ^6^ Seat at the Table representative Breast Cancer Network Australia Camberwell Victoria Australia; ^7^ Health Consumers New South Wales Sydney New South Wales Australia

**Keywords:** artificial intelligence, breast neoplasms, early detection of cancer, health services research, patient preference

## Abstract

**Background:**

Artificial intelligence (AI) could improve accuracy and efficiency of breast cancer screening. However, many women distrust AI in health care, potentially jeopardizing breast cancer screening participation rates. The aim was to quantify community preferences for models of AI implementation within breast cancer screening.

**Methods:**

An online discrete choice experiment survey of people eligible for breast cancer screening aged 40 to 74 years in Australia. Respondents answered 10 questions where they chose between two screening options created by an experimental design. Each screening option described the role of AI (supplementing current practice, replacing one radiologist, replacing both radiologists, or triaging), and the AI accuracy, ownership, representativeness, privacy, and waiting time. Analysis included conditional and latent class models, willingness‐to‐pay, and predicted screening uptake.

**Results:**

The 802 participants preferred screening where AI was more accurate, Australian owned, more representative and had shorter waiting time for results (all *p* < .001). There were strong preferences (*p* < .001) against AI alone or as triage. Three patterns of preferences emerged: positive about AI if accuracy improves (40% of sample), strongly against AI (42%), and concerned about AI (18%). Participants were willing to accept AI replacing one human reader if their results were available 10 days faster than current practice but would need results 21 days faster for AI as triage. Implementing AI inconsistent with community preferences could reduce participation by up to 22%.

## Abstract 2

Many Australian women have strong preferences against using AI in breast cancer screening, particularly without human oversight. Introducing AI without careful consumer engagement and tailored messaging is likely to excite some participants and alienate others, with potential equity implications.

## INTRODUCTION

Breast cancer screening programs remain a key component to improved breast cancer outcomes, offering a substantial reduction in mortality.^1^ In the United States, standard practice is for mammograms to be read by a single radiologist that may be supported with computer‐aided detection.^2^ However, in many population‐based programs worldwide, each mammogram image is reviewed independently by two readers (e.g., radiologists, radiographers), with a third reader or consensus meeting used for arbitration where necessary, to identify abnormalities requiring further assessment.^3^ This double reading approach improves cancer detection rates while keeping recall rates low.^4^, ^5^ However, it is resource intensive, and worldwide reader shortages mean sustainability is challenging.^6^

Artificial intelligence (AI) has the potential to improve the accuracy of breast cancer screening while also addressing reader shortages and reducing costs.^3^, ^6^, ^7^ However, evidence for the accuracy of AI is still emerging,^8^, ^9^, ^10^ with interim results from the first prospective randomized controlled trial recently published,^11^ and the best way to integrate AI into screening programs remains unclear.^8^ AI has been investigated to replace a reader in double reading screening programs (e.g., ^10^ ^,^ ^12^) or as a triage tool (e.g., ^9^ ^,^ ^13^), but there remains no universal consensus on the optimal service model(s) for implementing AI in breast cancer screening.

Cancer screening program success also depends on high rates of participation.^1^ Qualitative and descriptive studies suggest that although women are generally receptive to AI, many remain undecided and report being particularly unwilling to trust AI for their health care.^14^, ^15^, ^16^ And although dropout rates in trials of AI in breast cancer screening have been low,^11^ studies of women’s perspectives suggest introducing AI into breast cancer screening programs could jeopardize screening participation rates if participants do not trust AI.^16^, ^17^

Although qualitative and descriptive data are useful for informing policy, there are several advantages to quantitatively measuring patient or community preferences.^18^ These include the ability to investigate hypothetical situations that do not yet exist, evaluate competing policy options, analyze explicit tradeoffs people make, and investigate heterogeneity within the population to guide implementation.^18^ Discrete choice experiments (DCEs), based on the economic theory of random utility maximization,^19^ offer an innovative method to quantify the relative importance of different aspects of health and health care to consumers,^20^ and can also be used to model uptake of proposed interventions across the population.^20^, ^21^ There have been several DCE studies of radiologist preferences for the use of AI in breast cancer screening^22^, ^23^, ^24^; however, there are no published DCE studies of participant’s preferences for the use of AI in breast cancer screening.

The aim of this study is, for the first time, to quantitatively examine preferences for the use of AI in breast cancer screening among people eligible for breast cancer screening using an online DCE survey. We examine preferences for the role of AI (supplementing double reading, replacing one reader, triage, or AI alone), as well as AI accuracy, sovereignty, representativeness, privacy, and waiting time for the results. We then use the results to model breast screening uptake if AI was implemented in a range of policy‐relevant scenarios.

## MATERIALS AND METHODS

We administered a discrete choice experiment (DCE) survey to people eligible for breast cancer screening in Australia. The primary outcome was the relative importance of each attribute of breast cancer screening involving AI. Secondary outcomes were willingness to pay (measured with waiting time for results) and the predicted impact on uptake if AI were implemented in a variety of policy‐relevant scenarios.

### Survey development

**FIGURE 1:**
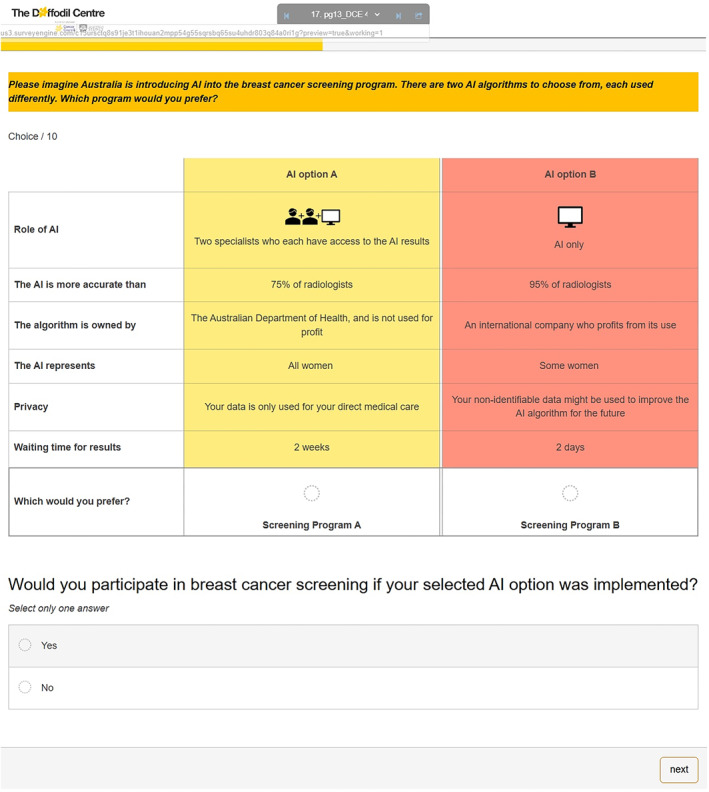
Example choice task within the discrete choice experiment survey.

The online survey instrument asked respondents to imagine that Australia is introducing AI into the breast cancer screening program. Respondents answered a series of 10 DCE choice questions. Each choice question offered a choice between two hypothetical screening programs using AI. The hypothetical screening programs in the choice questions were defined by six attributes (AI role, accuracy, sovereignty, representativeness, privacy, and waiting time), each with a range of varying levels (Table 1). Figure 1 presents an example DCE question in which each hypothetical screening program reflects one of the levels for each attribute.

**TABLE 1:** Attributes and levels for the discrete choice experiment.

Attribute	Levels
Role of AI	Two specialists who each have access to the AI results One specialist and one AI AI as triage – only those NOT at “very low risk” are examined by specialists AI only
Accuracy	The AI is more accurate than all radiologists The AI is more accurate than 95% of radiologists The AI is more accurate than 85% of radiologists The AI is more accurate than 75% of radiologists
Sovereignty	The AI is owned by an international company who profit from its use The algorithm is owned by an Australian company who profit from its use The algorithm is owned by the Australian Department of Health and is not used for profit
Representation in training and validation data	The AI represents all (100%) women The AI represents most (75%) women The AI represents about half (50%) of women
Privacy	Your data are only used for your direct medical care Your nonidentifiable data may be used to improve BreastScreen services or in research projects Your non‐identifiable data might be used to improve the AI algorithm for the future
Waiting time for results	2 days 1 week 2 weeks (current practice)

Abbreviation: AI, artificial intelligence.

Development of the survey followed good research practice guidelines,^25^, ^26^ including scoping reviews of the literature (see Supplementary Material 1 for details) and consultation with our Expert Collaborator Panel. Attributes were selected to provide relevant information to stakeholders for designing breast screening policy around the use of AI.

The attribute for the role of AI covered four options: supplementing current practice (double human reading) with AI, replacing one reader with AI, using AI as triage to identify 50% not at very low risk, and using AI alone. Although AI alone was considered an unlikely policy scenario, including it allowed us to capture the full spectrum of participant preferences by inducing tradeoffs between attributes.^27^ Accuracy of the AI was expressed in comparison to radiologists (who do all screening mammography reading in Australia), with levels varying from “AI is more accurate than 75% of radiologists” to “AI is more accurate than all radiologists.” This approach is consistent with a previous DCE among radiologists^24^ and minimizes cognitive burden for respondents in understanding concepts such as sensitivity and specificity. The levels reflect current evidence regarding the accuracy of AI as well as women’s perceptions of AI accuracy.^8^, ^9^, ^15^, ^28^, ^29^, ^30^ The levels of the remaining attributes can be seen in Table 1.

As well as asking participants to choose between the two options for AI in breast cancer screening, each choice question included a follow up asking whether they would participate in breast cancer screening if their selected program was rolled out. In addition, the survey instrument contained questions on respondent sociodemographic characteristics, cancer and screening history and knowledge, attitudes to AI,^31^ health literacy,^32^ risk tolerance,^33^ and comprehension of the DCE questions. The questions about knowledge of current breast cancer screening practices were designed to both collect self‐reported knowledge and inform participants of current practice.

The draft survey instrument was reviewed by the Expert Panel and piloted using cognitive interviews (Zoom or face‐to‐face, average 50 minutes) with a convenience sample of eight participants. Through iterative adjustments we confirmed the survey and choice task were easily understood and the attributes and levels were relevant.

The DCE choice questions were statistically designed to allow estimation of the main‐effect preference weights using a conditional logit model.^25^ A fractional factorial design was generated in NGENE v1.3 using the D‐efficiency criteria to select between competing designs. The experimental design included 32 choice questions, from which we created four blocks of eight choice questions. Respondents were randomly assigned to one block plus two additional choice questions inserted manually: a duplicate choice question to assess response consistency and a dominant choice question where one option was clearly better than the other to assess choice rationality.

There was no missing data because all questions were mandatory. Responses were checked for consistency, rationality, self‐reported understanding of the task, and completion time, but responses were not excluded based on these results.

### Study population

An online panel provider, PureProfile, recruited the sample. Inclusion criteria were people eligible for breast cancer screening, aged 40 to 74 years (breast cancer screening eligibility age in Australia). Exclusion criteria were those unable to complete the online survey due to cognitive, language, or logistical barriers. The estimated sample size required to obtain reliable estimates for preference data based on eight choice tasks, two alternatives per task, and six attributes with at most four levels, was 500, assuming estimation of main effects and two‐way interactions.^34^ However, we aimed for 800 responses to also allow exploratory subgroup analyses. Quotas were imposed for representativeness by age and State. All respondents provided electronic informed consent, and The University of Sydney Human Research Ethics Committee granted ethical approval for the survey and cognitive interviews (Ref 2023/437).

### Statistical analyses

The choice data were analyzed using conditional logit, mixed logit, and latent class models, consistent with good research practices.^25^ We present here the results of the conditional logit and latent class models (see Supplementary Material 2 for methods and results of mixed logit model), along with an analysis of predicted uptake. All attribute levels were dummy coded, except for accuracy and waiting time, which were analyzed as continuous variables.

The conditional logit model of main effects yields a preference weight (the coefficient) for the levels of each attribute. These preference weights represent the relative contribution of the attribute level to the utility that respondents assign to a hypothetical option^25^ and suggest the most important features to maximize uptake and participation in potential screening programs involving AI.

We further quantify preferences by calculating “willingness to pay” (and related confidence intervals) for the various features of AI in breast cancer screening. This is done by dividing the negative of the preference weight for each feature by the preference weight of a continuous attribute, in this case the waiting time for results. This allows us to estimate how long participants are willing to wait for their results to have a specific feature of AI. Cost was not included as an attribute because breast cancer screening is provided for free in Australia.

The latent class model uses the conditional logit model to yield the same preference weights, but assumes there are classes within the sample, with each class having systematically different preference weights.^25^ This allows exploration of patterns of preferences among potential subgroups in the population. Including potential explanatory sociodemographic characteristics in the latent class model tests the influence of individual characteristics on the probability of class membership.^25^ Three classes were optimal based on comparison of the model Akaike Information Criterion and Bayesian Information Criterion goodness‐of‐fit statistics.

The impact on predicted uptake of incorporating AI in breast cancer screening was assessed first through the average self‐reported participation if their selected AI option in each question was implemented. Second, for three alternative policy‐relevant scenarios for implementing AI in breast cancer screening (see Box 1), the indirect preference weights of the combined attribute levels was used to estimate average individual uptake for each option and compared to the base case (where all preference weights are assumed to be 0).^21^ This analysis was limited to respondents who had previously participated in breast cancer screening (*n* = 531) to reflect changes in screening behavior because absolute predicted participation rates were unlikely to reflect uptake at a population level.

**BOX 1**

1. **Scenario 1:** Supplementing current practice with AI (i.e., double human reading both with access to AI results) compared to replacing one human in double human reading with AI (i.e., one human + one AI read).
2. **Scenario 2:** AI implemented only once evidence of high levels of accuracy were available (the AI is more accurate than 100% of radiologists), as women have been shown to expect in qualitative studies^15^, ^28^, ^29^ compared to implementing AI at current levels of accuracy, assumed to be that the AI is more accurate than 62% of radiologists, consistent with current literature.^30^
3. **Scenario 3:** Implementing an international algorithm that is representative of most women (75%) compared to implementing a locally developed algorithm that is representative of all women (100%).

Unless otherwise noted, all scenarios assumed the algorithm replaced one radiologist, had accuracy >90% of radiologists, international for‐profit company ownership, representative of most women, current practice for privacy, and 7‐day wait time for results.

AI, artificial intelligence.

All analyses were completed in STATA (v15.1), using the clogit, wtp, mixlogit, glam, and lclogit commands. A post hoc analysis to control the false discovery rate using the Benjamini‐Hochberg adjustment^35^ was applied to the conditional logit and latent class models. The study datasets generated and analyzed are available from the corresponding author on reasonable request.

## RESULTS

There were 802 completed responses (from 918 starts: 13 screened out, 103 timed out) to the online DCE survey. The sample was generally representative of the Australian population of people eligible for breast cancer screening in terms of age, state, and remoteness. Two thirds (66%) had previously participated in breast cancer screening, with 22% previously recalled, and 5% previously diagnosed with breast cancer. Knowledge of breast cancer screening practices was low, with only 21% of participants aware that double reading with arbitration currently takes place. See Table 2 for further sociodemographic details and Supplementary Material 3 for details by previous screening status.

**TABLE 2:** Sociodemographic details of discrete choice experiment survey participants.

	Total (*N* = 802)
Gender	
Female	801 (100%)
Other	1 (<1%)
Age (years)	
40–49	243 (30%)
50–59	247 (31%)
60–69	227 (28%)
70–74	85 (11%)
State	
New South Wales	220 (27%)
Victoria	210 (26%)
Queensland	166 (21%)
Western Australia	81 (10%)
Northern Territory	1 (<1%)
South Australia	80 (10%)
Tasmania	35 (4%)
Australian Capital Territory	9 (1%)
Urban/rural	
Major city	452 (56%)
Regional	276 (34%)
Rural/remote	72 (9%)
Unknown	2 (<1%)
Overall health	
Excellent	47 (6%)
Very good	203 (25%)
Good	343 (43%)
Fair	173 (22%)
Poor	36 (4%)
Ever participated in breast cancer screening?	
Yes	531 (66%)
No	256 (32%)
Unsure	15 (2%)
Ever participated other cancer screening?	
Yes	507 (63%)
No	286 (36%)
Unsure	9 (1%)
Ever been recalled after breast cancer screening?	
Yes	180 (22%)
No	345 (43%)
Unsure	6 (1%)
Missing	271 (34%)
Ever diagnosed with breast cancer?	
Yes	39 (5%)
No	760 (95%)
Unsure	3 (<1%)
Did you know you are eligible and invited to BreastScreen between the ages of 50 and 74 years?	
Yes	672 (84%)
No	91 (11%)
Some, but not all	39 (5%)
Did you know that two radiologists review each mammogram image, and if they disagree then a third radiologist opinion is sought (called arbitration)?	
Yes	166 (21%)
No	545 (68%)
Some, but not all	91 (11%)
Did you know that for every 10,000 women screened and who have no cancer detected, approximately eight or nine will be diagnosed with a breast cancer in the next 2 years. This could be because it is a very aggressive cancer, or because it was “missed” by the breast cancer screening.	
Yes	156 (19%)
No	507 (63%)
Some, but not all	139 (17%)
Did you know that for every 10,000 women screened, approximately 500 are recalled (asked to return) for further assessments because of an abnormal screening mammogram? Approximately 60 of these people will go on to be diagnosed with breast cancer	
Yes	131 (16%)
No	505 (63%)
Some, but not all	166 (21%)
Did you know the average wait time for results through BreastScreen is about 2 weeks?	
Yes	357 (45%)
No	382 (48%)
Some, but not all	63 (8%)

Attitudes to AI^31^ were overall neutral, with an average of 6/11 and 5/11 for the AI Fear and Acceptance subscales, respectively. Most respondents (85%) reported they understood the concept of making a choice within the DCE section, 91% passed the dominance test of validity, and 74% passed the duplicate choice test of attention (only 3% failed both, and these respondents were kept in the analysis).

### Preferences

Overall, the conditional logit model shows participants preferred screening programs where the AI was more accurate (*p* < .001), owned by an Australian company (*p* < .001) or Department of Health (*p* < .001), was more representative (*p* < .01), and had a shorter waiting time for results (*p* < .001) (Table 3). There was a strong preference against screening programs which relied on AI alone (*p* < .001), whereas privacy was the only factor that did not significantly impact participant preferences (*p* = .992 and *p* = .372).

**TABLE 3:** Conditional logit model of preferences and attribute relevant importance.

Attributes	Coefficient	*p* value	Adjusted *p* value*	95% CI	
Role of AI (base = 2 human readers who both have access to AI results)					
One specialist + one AI	–0.300	<.001	<.002	–0.390	–0.210
AI as triage	–0.628	<.001	<.002	–0.738	–0.517
AI only	–1.153	<.001	<.002	–1.284	–1.023
AI accuracy (more accurate than x% of radiologists)					
Accuracy	0.022	<.001	<.002	0.019	0.026
Algorithm ownership (base = The Australian Department of Health and is not used for profit)					
An international company who profit from its use	–0.840	<.001	<.002	–0.955	–0.725
An Australian company who profit from its use	–0.286	<.001	<.002	–0.375	–0.197
AI representation (base = all women)					
Most women	–0.102	.006	<.01	–0.174	–0.030
Some women	–0.231	<.001	<.002	–0.315	–0.147
Privacy (base = direct medical care only)					
Improve BreastScreen or research	0.000	.992	.992	–0.082	0.081
Improve AI algorithm	0.034	.372	.409	–0.041	0.110
Waiting time for results (days)					
Waiting time	–0.030	<.001	<.002	–0.036	–0.023

*Note*: Log pseudolikelihood = –3793.27; pseudo *R* ^2^ = 0.4170. *False discovery rate *p* value based on false discovery rate of 5%, calculated post‐hoc using the Benjamini‐Hochberg correction for multiple hypothesis testing.

Abbreviations: AI, artificial intelligence; CI, confidence interval.

**FIGURE 2:**
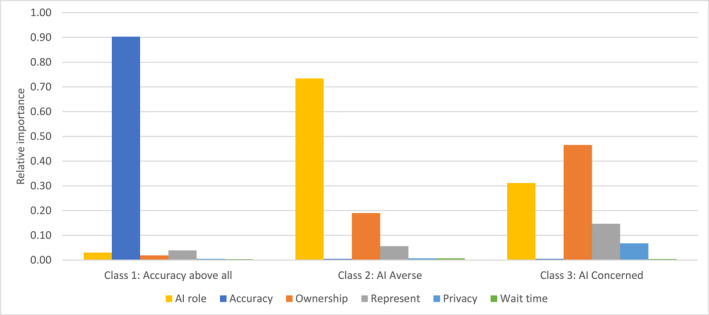
Latent class analysis results displayed as relative importance by class.

Three distinct patterns of preferences emerge among participants in the latent class analysis (see Figure 2 and Table 4). Class 1 (40% of the sample) we call “accuracy above all” as accuracy is the most important attribute for them. This group does not have strong preferences around how AI is used or ownership, although representativeness is somewhat important. Participants in this class are more likely to have previously participated in breast cancer screening.

**TABLE 4:** Latent class analysis results.

	Coefficient	*p* value	Adjusted *p* value*	95% CI		
Class 1 – accuracy above all (class share = 40%)						
Role of AI (base = 2 human readers who both have access to AI results)						
One specialist + one AI	–0.350	<.001	<.003	–0.525	–0.174	
AI as triage	–0.169	.069	.093	–0.352	0.013	
AI only	–0.097	.411	.491	–0.327	0.134	
AI accuracy (more accurate than x% of radiologists)						
Accuracy	0.034	<.001	<.003	0.028	0.040	
Algorithm ownership (base = The Australian Department of Health, and is not used for profit)						
An international company who profits from its use	–0.219	.008	.016	–0.380	–0.057	
An Australian company who profits from its use	–0.019	.799	.859	–0.162	0.125	
AI representation (base = all women)						
Most women	–0.298	<.001	<.003	–0.430	–0.165	
Some women	–0.452	<.001	<.003	–0.604	–0.301	
Privacy (base = direct medical care only)						
Improve BreastScreen or research	0.007	.923	.944	–0.126	0.139	
Improve AI algorithm	0.053	.462	.537	–0.088	0.194	
Waiting time for results (days)						
Waiting time	–0.043	<.001	<.003	–0.054	–0.032	
Class 2 – AI averse (class share = 42%)						
Role of AI (base = 2 human readers who both have access to AI results)						
One specialist + one AI	–0.417	<.001	<.003	–0.625	–0.210	
AI as triage	–1.387	<.001	<.003	–1.701	–1.072	
AI only	–2.957	<.001	<.003	–3.407	–2.507	
AI accuracy (more accurate than x% of radiologists)						
Accuracy	0.019	<.001	<.003	0.011	0.027	
Algorithm ownership (base = The Australian Department of Health and is not used for profit)						
An international company who profits from its use	–0.766	<.001	<.003	–0.982	–0.550	
An Australian company who profits from its use	–0.197	.042	.060	–0.386	–0.007	
AI representation (base = all women)						
Most women	–0.105	.283	.348	–0.296	0.087	
Some women	–0.226	.025	.040	–0.425	–0.028	
Privacy (base = direct medical care only)						
Improve BreastScreen or research	–0.013	.891	.934	–0.196	0.170	
Improve AI algorithm	–0.029	.772	.851	–0.225	0.167	
Waiting time for results (days)						
Waiting time	–0.028	<.001	<.003	–0.042	–0.014	
Class 3 – AI concerned (class share = 18%)						
Role of AI (base = 2 human readers who both have access to AI results)						
One specialist + one AI	–5.251	.005	.011	–8.903	–1.598	
AI as triage	–12.997	.006	.013	–22.245	–3.748	
AI only	–9.003	.003	.007	–14.883	–3.123	
AI accuracy (more accurate than x% of radiologists)						
Accuracy	–0.191	.016	.028	–0.346	–0.036	
Algorithm ownership (base = The Australian Department of Health and is not used for profit)						
An international company who profit from its use	–19.417	.002	.005	–31.989	–6.845	
An Australian company who profit from its use	–6.119	.002	.005	–10.058	–2.181	
AI representation (base = all women)						
Most women	–1.273	.015	.027	–2.302	–0.244	
Some women	–6.121	.015	.027	–11.040	–1.203	
Privacy (base = direct medical care only)						
Improve BreastScreen or research	1.031	.071	.093	–0.088	2.150	
Improve AI algorithm	–1.792	.088	.111	–3.849	0.265	
Waiting time for results (days)						
Waiting time	–0.176	.029	.045	–0.334	–0.018	
Class membership model parameters (Class 2 – AI averse = reference class)						
Class 1 – accuracy above all						
Age	0.008	.944	.944	–0.228	0.245	
Previous breast cancer screening	0.768	.001	<.003	0.309	1.227	
Fear of AI	–0.214	<.001	<.003	–0.320	–0.108	
Number of opt outs	–0.088	.023	.038	–0.164	–0.012	
Constant	0.290	.700	.792	–1.185	1.764	
Class 3 – AI concerns						
Age	0.497	<.001	<.003	0.230	0.764	
Previous breast cancer screening	0.583	.041	.060	0.024	1.143	
Fear of AI	–0.229	.001	<.003	–0.362	–0.096	
Number of opt outs	–0.092	.061	.085	–0.188	0.004	
Constant	–2.261	.010	.020	–3.981	–0.540	

*Note*: Mean highest posterior probability = 0.8625. *False discovery rate *p* value based on false discovery rate of 5%, calculated post‐hoc using the Benjamini‐Hochberg correction for multiple hypothesis testing.

Abbreviations: AI, artificial intelligence; CI, confidence interval.

Class 2 (42% of the sample) we call “AI averse” as they prefer AI to supplement current practice rather than to replace a reader, be used as triage, or used alone, although this is not as strong a preference as Class 3. They prefer Australian owned and not‐for‐profit companies, but representativeness is not a significant consideration. Participants in this class were more likely to report elevated fear of AI, and the most likely to say they would not participate in breast cancer screening if AI was implemented. They were also least likely to have previously participated in breast cancer screening, although they were not different in age from those in Class 1.

Class 3 (18% of the sample) we call “AI concerned” because they have strong preferences against algorithms owned by an international company and are the most likely to be concerned about representativeness of the algorithm. They are against all roles of AI but have very strong preferences against AI as triage. Participants in this class are older than the other classes.

### Willingness to pay

**FIGURE 3:**
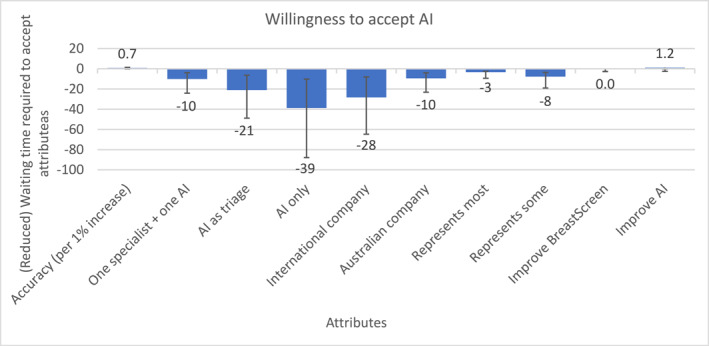
Willingness to accept artificial intelligence, using waiting time for results as the measure of “payment.”

The willingness to pay results (Figure 3) show participants would be willing to accept AI replacing one human reader if their results were available 10 days faster than double human reading. They would need the results to be available 21 days faster for AI as triage and 39 days faster for AI only. Given that the current waiting time is 14 days, this suggests a strong aversion to AI as triage or AI alone. Participants also had a strong preference for the algorithm to be owned by the Australian government and not used for profit; they were willing to wait an additional 28 days rather than have an algorithm owned by an international, for‐profit, company. Participants were willing to wait an additional week to improve accuracy by 5%, and a similar time (8 days) to ensure the algorithm was representative of all women.

### Uptake

On average, respondents indicated that they would not participate in breast cancer screening if their selected AI option was implemented in 1.9 (of 8) choice questions. Although 57% of participants indicated they would always participate, 12% indicated they would never participate. Always choosing “no” was more common (average 2.4) among those who had not previously participated in breast cancer screening.

Among participants who had previously participated in breast cancer screening (*n* = 531) we see that, in scenario 1, introducing AI as a replacement for one reader reduced predicted screening uptake by 5.3% compared to supplementing current double human reading with the AI results. In scenario 2, introducing a locally developed algorithm that is accurate for all women, rather than an internationally owned algorithm that is representative for most women improved uptake by 18.1%. Finally, in scenario 3, participants were 21.9% less likely to take up screening if AI was introduced with current rates of accuracy (assuming AI is more accurate than 62% of radiologists, based on Rodriguez‐Ruiz 2019^30^), compared to AI being introduced at a point when it is more accurate than 100% of radiologists (based on qualitative findings suggesting this is women’s expectations^15^, ^28^, ^29^).

## DISCUSSION

This large, representative survey of people eligible for breast cancer screening in Australia suggests participants preferred AI to be implemented within screening programs when the algorithm is more accurate, locally owned, representative of the population, and reduces waiting times. It also indicates there are strong preferences against the use of AI alone or as triage. Interestingly, how data were used (privacy) did not significantly impact participant preferences.

This is consistent with existing qualitative and descriptive literature, which suggests women are generally receptive to the benefits of AI in breast cancer screening^15^ but feel strongly that AI should supplement or support clinicians. For example, more than three quarters of survey respondents agreed or strongly agreed that a human check is required for AI to be used in breast cancer screening in the Netherlands^14^ and 94% felt a radiologist should always report on mammograms regardless of AI use in Italy.^36^ Similar results are seen outside the context of breast cancer screening, with a human second reader regarded as useful by patients considering AI in general radiology,^28^ and 94% of participants wanting symbiosis between AI and clinicians for skin cancer screening.^37^

Three distinct patterns of preferences were seen among our respondents. Although 18% of the sample expressed concerns about the specifics of AI within breast cancer screening, such as representation and ownership, the remaining were equally split (∼40% each) between accepting AI if it improved accuracy or being highly averse to AI. Similar diversity in responses was seen in a survey of women in the Netherlands, where 42% of women disagreed that AI should be used as triage, while 32% agreed.^14^ These results suggest there is a real risk that introducing AI without careful consumer engagement and tailored messaging would excite some participants and alienate others, with potential equity implications. Given the diversity of views in the population, making policy decisions based on the aggregation of individual preferences will be difficult. A recent national citizens’ jury on the use of AI in health care^38^ highlights the potential to support members of the public to come to consensus on what is best for everyone on these contested questions.

The value of accuracy was previously described in a qualitative focus group study in Australia.^29^ Accuracy was extremely important to 80% to 95% of women eligible for breast cancer screening, and women expected AI systems to have strong evidence they perform better than current systems before implementation.^29^ This is consistent with participants in other studies who believed that AI would not be implemented without clear evidence of accuracy or effectiveness.^15^, ^28^ The value of the DCE method is that it allows us to identify how strong this preference is, and in fact we see that for 40% of the population, accuracy is their primary consideration, whereas for the remaining 60%, the implementation of AI is of greater concern.

The importance to participants of representation with the training and validation data is a striking result. Focus groups with women of screening age have found that potential discriminatory bias is one of the main concerns expressed by women in the United Kingdom about AI in breast cancer screening,^15^ and that it was considered a “given” that the AI would treat everyone fairly among women in Australia.^29^ However, this is the first study that has been able to quantify the sizeable proportion of people eligible for breast cancer screening (18%) for whom this forms the basis of their preferences for how AI is implemented in practice.

Views about AI in breast cancer screening may impact participation. Our results identified the potential impact of various implementation approaches on uptake rates. Given the generally positive views seen in the existing literature around the use of AI in conjunction with a human reader,^15^, ^28^, ^37^ the relatively small reduction in participation when moving from AI supplementing current practice to AI replacing one radiologist (5.3%) is understandable.

However, there is a disparity between women’s expectations for the accuracy of AI before implementation and the current reality.^15^, ^28^, ^29^ In our results, we see that adoption of AI within breast cancer screening at current rates of accuracy could reduce participation by >20% rather than waiting. This is in keeping with the recommendations of Hofmann^39^ that health system decision makers should deploy new technologies judiciously, and the recommendation of Carter et al^29^ that conservative decision making about implementation and clear communication about performance of any AI system will be required to meet the communities high expectations in the context of breast cancer screening.

Ownership of data used by algorithms is a concern for all areas of AI and particularly health.^40^, ^41^ In relation to AI in breast cancer screening, concerns over data safety, security, and confidentiality were mentioned but less discussed in focus groups, including the involvement of large companies such as Google and Facebook.^29^ Our results confirm that introduction of a locally developed algorithm, which has benefits of representation of the population in training and development would improve uptake by 18% compared to implementation of an international algorithm that is representative of most women. Although Australia currently has regulatory approval for only one algorithm for breast cancer detection,^42^ there are several currently in development locally (e.g., ^43^ ^,^ ^44^), and health care decision makers may benefit from providing additional support to these.

While this is the first quantitative study examining preferences for the use of AI in breast cancer screening among people eligible for breast cancer screening, there are some limitations. Our study is representative of Australian women; however, attitudes to AI may differ in contexts with different social values or existing screening practices. We provided participants with information about AI and how it can be used in breast cancer screening, which may have changed reported preferences; we saw that few participants were aware of current double‐reading practices, and previous work shows that attitudes can change following information or discussion about AI.^29^, ^45^ And finally, although discrete choice experiments offer a unique and valuable method to quantify patient preferences, anchoring bias may reduce the reliability of the willingness to pay outcomes, and hypothetical bias may mean that stated preferences do not reflect true preferences as patients are unlikely to be offered this choice in practice.^20^, ^21^

## CONCLUSIONS

Although AI may improve effectiveness and/or efficiency of mammography reading in breast cancer screening, these benefits may be offset if screening participation is reduced. This is particularly concerning in Australia, where participation rates in BreastScreen are already relatively low.^46^ Implementing AI in breast cancer screening will need careful communication to screening populations around the potential risks and benefits, particularly for issues such as how the AI will be implemented, the synergies between the AI and human readers, the accuracy of the algorithm, how representative the underlying algorithm is of the general population, and who owns the algorithm and associated data.

## AUTHOR CONTRIBUTIONS

**Alison Pearce:** Conceptualization; methodology; formal analysis; funding acquisition; investigation; project administration; writing—original draft; writing—review and editing. **Stacy Carter, Helen M.L. Frazer, Nehmat Houssami, Mary Macheras‐Magias, and Genevieve Webb:** Assisted with methodology and conceptualization and writing—review and editing. **M. Luke Marinovich:** Methodology; conceptualization; funding acquisition; writing—review and editing. All authors read and approved the final manuscript.

## CONFLICT OF INTEREST STATEMENT

Nehmet Houssami declares membership of Australia’s Therapeutic Goods Administration (TGA) Advisory Committee on Medical Devices. All other authors declare no financial or nonfinancial conflicts of interests.

## PATIENT CONSENT STATEMENT

Informed consent was obtained from all individual participants included in the study.

## Supporting information

Supplementary Material

## Data Availability

The data that support the findings of this study are available on request from the corresponding author. The data are not publicly available because of privacy and ethical restrictions.
