# Supplementary material for: Implementing artificial intelligence in breast cancer screening: Women’s preferences
Source: Cancer. 2025 Apr 22;131(9):e35859. doi: 10.1002/cncr.35859 (PMC12013981; doi:10.1002/cncr.35859)
Supplement: Supplementary file 1 — Supplementary Material [file CNCR-131-e35859-s001.docx]

**Supplementary Material**

**Implementing artificial intelligence in breast cancer screening: women’s preferences**

Alison Pearce^1,2^, Stacy Carter^3^, Helen ML Frazer^4,5^, Nehmat Houssami^1,2^, Mary Machera-Magis^6^, Genevieve Webb^7^, M. Luke Marinovich^1,2^

^1^ The Daffodil Centre, The University of Sydney, a joint venture with Cancer Council New South Wales

^2^ Sydney School of Public Health, The University of Sydney

^3^ Australian Centre for Health Engagement, Evidence and Values, School of Health and Society, University of Wollongong

^4^ St Vincent’s Hospital Melbourne

^5^ BreastScreen Victoria

^6^ Breast Cancer Network Australia, Seat at the Table representative

^7^ Health Consumers New South Wales representative

**Supplementary Material 1: Summary of scoping reviews methods and results**

Two structured scoping reviews of the literature around preferences for the use of AI in disease screening and diagnosis were conducted. The first review identified previous discrete choice experiments conducted to establish preferences of clinicians, women or the community for the use of AI in screening for any cancer type. The second review included both qualitative and quantitative studies (but not discrete choice experiments) that examined women’s preferences for the use of AI in breast cancer screening.

Searches were conducted in PubMed (Medline), Embase and Scopus (example full search strategies can be seen in Table A). In summary, the search terms were identified from previous successful reviews to cover the concepts of:

- artificial intelligence, taken from two recent systematic reviews of economic evaluations of artificial intelligence in health care [1,2],
- discrete choice experiments, based on a recent systematic review of discrete choice experiments in health care [3],
- consumer preference, values and beliefs, based on a recent systematic review of women’s values and preferences for breast cancer screening and diagnostic services [4].
- Breast cancer [4].

The review of previous discrete choice experiments found three DCEs examining the preferences of clinicians for the use of AI in breast cancer screening [5–7]. There were no DCEs identified conducted with women about their preferences for the use of AI in breast cancer screening, or with any group for preferences relating to the use of AI in other cancer types. Two of these studies focussed on the attributes of the algorithm, such as sensitivity, specificity, supporting evidence, validation and diversity of training data [5,6], while the third focussed on attributes related to implementation, such as time savings and price [7]. Clinicians were found to highly value sensitivity and the participation of radiologists in interpretation, and to be more positive about the role of AI in routine diagnostics, rather than as a tool to reduce scan time. This highlights a gap in the literature for an examination of women’s preferences.

The review of women’s values, attitudes and beliefs (non-DCE studies) found 5 relevant studies [8–12], although none were conducted in Australia. Overall, the results suggest that while women believe AI can improve speed, efficiency and accuracy of breast cancer screening [8,11,12] there is a general lack of awareness, knowledge and understanding of AI and how it can apply to healthcare [10–12]. While women are generally happy for AI to be used to supplement or assist radiologists with decision making [10], they are often uncertain about AI replacing a radiologist for mammogram reading [9–11]. There is a lack of trust among women in relation to the use of AI for breast cancer screening, particularly in relation to data privacy [11,12]. Women are also concerned about discriminatory bias of AI [12], and want human oversight of the AI and human interaction with patients [8,11,12].

The results of the review(s) were used to develop a ‘long list’ of potential attributes for inclusion in the DCE, highlighting those attributes that are indicated in the literature as most likely to be important to consumers. The identified attributes fell into six main categories: Accuracy of the AI, Radiologist involvement in the process, Development of the AI, Efficiency of the AI, Risks of the AI, Costs. A small number of additional attributes were identified which did not fit into any of these categories. The longlist is presented in Table B.

**Table A: Search strategy**

*Example search strategy for discrete choice experiment evidence:*

PubMed (Medline) 28^th^ August 2022

1. Artificial intelligence [Title/Abstract]
2. Artificial intelligence [Mesh]
3. 1 OR 2
4. Machine learning [Title/Abstract]
5. Machine learning [Mesh]
6. 4 OR 5
7. Deep learning [Title/Abstract]
8. Computer aided [Title/Abstract]
9. Computer-aided design [Mesh]
10. 8 OR 9
11. CAD [Title/Abstract]
12. Data driven [Title/Abstract]
13. 3 OR 6 OR 7 OR 10 OR 11 OR 12
14. Discrete choice experiment* [Title/Abstract]
15. Discrete choice modeling [Title/Abstract] OR discrete choice modelling [Title/Abstract]
16. Discrete conjoint experiment [Title/Abstract]
17. Stated preference* [Title/Abstract]
18. Part-worth utilities [Title/Abstract]
19. Functional measurement [Title/Abstract]
20. Paired comparison* [Title/Abstract]
21. Pairwise choices [Title/Abstract]
22. Conjoint analysis [Title/Abstract]
23. Conjoint measurement [Title/Abstract]
24. Conjoint studies [Title/Abstract]
25. Conjoint choice experiment* [Title/Abstract]
26. 14 OR 15 OR 16 OR 17 OR 18 OR 19 OR 20 OR 21 OR 22 OR 23 OR 24 OR 25
27. 13 AND 26
28. Filter: Medline

*Example search strategy for qualitative evidence:*

PubMed (Medline) 28^th^ September 2022:

1. Breast Neoplasms [MESH]
2. Breast [Title/Abstract]
3. 1 OR 2
4. Mass Screening [MESH]
5. Early Detection of Cancer [MESH]
6. screen* [Title/Abstract]
7. 4 OR 5 OR 6
8. 3 AND 7
9. Mammography [MESH]
10. mammogram* [Title/Abstract]
11. 9 OR 10
12. diagnos* OR overdiagnos* OR over diagnos* OR overdetection OR over detection [Title/Abstract]
13. 3 AND 12
14. 8 OR 11 OR 13
15. Artificial intelligence [Title/Abstract]
16. Artificial intelligence [Mesh]
17. 15 OR 16
18. Machine learning [Title/Abstract]
19. Machine learning [Mesh]
20. 18 OR 19
21. Deep learning [Title/Abstract]
22. Computer aided [Title/Abstract]
23. Computer-aided design [Mesh]
24. 22 OR 23
25. CAD [Title/Abstract]
26. Data driven [Title/Abstract]
27. 17 OR 20 OR 21 OR 24 OR 25 OR 26
28. Choice Behavior [MESH]
29. Decision Making [MESH]
30. Attitude to Health [MESH]
31. Understanding OR Perception* OR Preference* OR Attitude* OR Expectation* OR Value* OR View* OR Informed decision* [Title/Abstract]
32. 28 OR 29 OR 30 OR 31
33. Qualitative research [MESH]
34. Focus Groups [MESH]
35. Qualitative OR interview* OR focus group* OR purposive OR grounded theory [Title/Abstract]
36. 33 OR 34 OR 35
37. 32 OR 36
38. 14 AND 27 AND 37
39. Filter: Medline

**Table B: Longlist of attributes identified in the literature**

| **Attribute** | **Source** |
| --- | --- |
| ***ACCURACY*** |  |
| Sensitivity | DCE: [5] |
| Specificity | DCE: [5] |
| Performance (sensitivity & specificity combined) | DCE: [6] |
| Quality – same as or better than radiologist | DCE: [7] |
| Accuracy | Descriptive: [8,12] |
| Consistency & objectivity | Descriptive: [8,12] |
| Reliability | Descriptive: [11,12] |
| Safety | Descriptive: [12] |
| ***RADIOLOGIST INVOLVEMENT*** |  |
| Role of AI - Complement/replace radiologists with AI, triage, reporting companion, hanging protocol, triage workflow | DCE: [5]  Descriptive: [8–11] |
| Need for human involved / checking | Descriptive: [9–11] |
| Human interaction & communication & empathy | Descriptive: [8,11,12] |
| ***DEVELOPMENT OF AI*** |  |
| Understandability of AI decision making (clinicians, AI experts, no one can understand algorithm) | DCE: [5] |
| Supporting evidence and algorithm validation | DCE: [5,6]  Descriptive: [11] |
| Diversity of training data, potential discriminatory bias | DCE:[5]  Descriptive: [12] |
| ***EFFICIENCY*** |  |
| Time saving (faster processing, diagnostic speed) | DCE: [7]  Descriptive: [8,11] |
| Improved efficiency | Descriptive: [8,11,12] |
| Convenience | Descriptive: [8] |
| ***RISKS and BENEFITS OF AI*** |  |
| Privacy | Descriptive: [8,12] |
| Responsibility for errors | Descriptive: [9,11] |
| Trust | Descriptive: [11] |
| Governance | Descriptive: [12] |
| Nefarious use of AI | Descriptive: [8] |
| Human deskilling | Descriptive: [8] |
| ***COSTS*** |  |
| Price per study | DCE: [7] |
| Reduced health care costs | Descriptive: [8,11,12] |
| ***OTHER*** |  |
| How suspicion of malignancy is communicated (binary, categorical, probability) | DCE: [5] |
| Provider (manufacturer, software co, AI startup) | DCE: [7] |
| Application (diagnostics, process efficiency, screening) | DCE: [7] |
| Patient anxiety | Descriptive: [8] |
| Patient education | Descriptive: [8] |

**Supplementary Material 2: Methods and results of mixed logit model**

While DCEs are commonly analysed using the conditional logit model, it does not allow preference heterogeneity to be assessed. To overcome this limitation, a mixed-logit model (and latent class analysis) was also conducted. The mixed-logit model yields both a mean and standard deviation effect across the sample, reflecting the differences in preferences among respondents. The results are interpreted by examining the statistical significance of the standard deviations for each attribute, and where this is <0.05 it indicates there is significant heterogeneity.

Our results (Table C) suggest there is significant heterogeneity in participant preferences in relation to the use of AI as triage or AI only, the ownership of the algorithm by an international company, and whether an individual’s results are used to improve BreastScreen or for future research. From the magnitudes of the standard deviations relative to the to the mean coefficients, we see that while only 1% of participants prefer AI as triage, 11% prefer AI only. We also see that 13% of participants prefer ownership by an international company, and 51% do not want their data to be used to improve BreastScreen or for research.

| **Table C: Mixed logit results** | | | | |
| --- | --- | --- | --- | --- |
| **Choice** | **Coef.** | **P>z** | **95% Conf. Interval** | |
| **Mean** |  |  |  |  |
| **Role of AI** *(base = 2 human readers who both have access to AI results)* | | | | |
| One specialist + one AI | -0.328 | 0.000 | -0.435 | -0.222 |
| AI as triage | -0.759 | 0.000 | -0.880 | -0.638 |
| AI only | -1.481 | 0.000 | -1.647 | -1.315 |
| **AI accuracy** *(more accurate than x% of radiologists)* | | | | |
| Accuracy | 0.027 | 0.000 | 0.023 | 0.031 |
| **Algorithm ownership** *(base = The Australian Department of Health, not used for profit)* | | | | |
| An international company who profit from its use | -1.094 | 0.000 | -1.226 | -0.963 |
| An Australian company who profit from its use | -0.387 | 0.000 | -0.486 | -0.288 |
| **AI representation** *(base = All women)* |  |  |  |  |
| Most women | -0.178 | 0.000 | -0.275 | -0.081 |
| Some women | -0.324 | 0.000 | -0.431 | -0.218 |
| **Privacy (base = Direct medical care only)** |  |  |  |  |
| Improve BreastScreen or research | 0.009 | 0.851 | -0.086 | 0.105 |
| Improve AI algorithm | 0.028 | 0.572 | -0.068 | 0.123 |
| **Waiting time for results (days)** |  |  |  |  |
| Waiting time | -0.035 | 0.000 | -0.043 | -0.028 |
|  |  |  |  |  |
| **Standard deviation** |  |  |  |  |
| **Role of AI (base = 2 human readers who both have access to AI results)** | | | | |
| One specialist + one AI | 0.279 | 0.077 |  |  |
| AI as triage | 0.343 | 0.018 |  |  |
| AI only | 1.194 | 0.000 |  |  |
| **Algorithm ownership (base = The Australian Department of Health, not used for profit)** | | | | |
| An international company who profit from its use | 0.989 | 0.000 |  |  |
| An Australian company who profit from its use | 0.031 | 0.797 |  |  |
| **AI representation (base = All women)** |  |  |  |  |
| Most women | 0.074 | 0.515 |  |  |
| Some women | 0.043 | 0.891 |  |  |
| **Privacy (base = Direct medical care only)** |  |  |  |  |
| Improve BreastScreen or research | 0.300 | 0.008 |  |  |
| Improve AI algorithm | 0.019 | 0.858 |  |  |

**Supplementary Material 3: Sociodemographic details of the sample, by previous screening status**

|  |  | **Previous participation in breast cancer screening** | | |  |
| --- | --- | --- | --- | --- | --- |
|  | **Total** | **Yes** | **No** | **Unsure** | **p-value** |
|  | N=802 | N=531 | N=256 | N=15 |  |
| **Gender** |  |  |  |  | <0.001 |
| Female | 801 (100%) | 531 (100%) | 256 (100%) | 14 (93%) |  |
| Other | 1 (0%) | 0 (0%) | 0 (0%) | 1 (7%) |  |
|  |  |  |  |  |  |
| **Age** |  |  |  |  | <0.001 |
| 40-49 | 243 (30%) | 88 (17%) | 146 (57%) | 9 (60%) |  |
| 50-59 | 247 (31%) | 177 (33%) | 66 (26%) | 4 (27%) |  |
| 60-69 | 227 (28%) | 189 (36%) | 36 (14%) | 2 (13%) |  |
| 70-74 | 85 (11%) | 77 (15%) | 8 (3%) | 0 (0%) |  |
|  |  |  |  |  |  |
| **State** |  |  |  |  | 0.52 |
| NSW | 220 (27%) | 142 (27%) | 74 (29%) | 4 (27%) |  |
| Vic | 210 (26%) | 132 (25%) | 74 (29%) | 4 (27%) |  |
| Qld | 166 (21%) | 117 (22%) | 48 (19%) | 1 (7%) |  |
| WA | 81 (10%) | 56 (11%) | 21 (8%) | 4 (27%) |  |
| NT | 1 (0%) | 0 (0%) | 1 (0%) | 0 (0%) |  |
| SA | 80 (10%) | 55 (10%) | 23 (9%) | 2 (13%) |  |
| Tas | 35 (4%) | 22 (4%) | 13 (5%) | 0 (0%) |  |
| ACT | 9 (1%) | 7 (1%) | 2 (1%) | 0 (0%) |  |
|  |  |  |  |  |  |
| **Remote** |  |  |  |  | 0.72 |
| Major city | 452 (56%) | 299 (56%) | 143 (56%) | 10 (67%) |  |
| Regional | 276 (34%) | 178 (34%) | 93 (36%) | 5 (33%) |  |
| Rural/remote | 72 (9%) | 53 (10%) | 19 (7%) | 0 (0%) |  |
| Unknown | 2 (0%) | 1 (0%) | 1 (0%) | 0 (0%) |  |
|  |  |  |  |  |  |
| **Health** |  |  |  |  | 0.47 |
| Excellent | 47 (6%) | 29 (5%) | 18 (7%) | 0 (0%) |  |
| Very good | 203 (25%) | 143 (27%) | 56 (22%) | 4 (27%) |  |
| Good | 343 (43%) | 228 (43%) | 110 (43%) | 5 (33%) |  |
| Fair | 173 (22%) | 112 (21%) | 56 (22%) | 5 (33%) |  |
| Poor | 36 (4%) | 19 (4%) | 16 (6%) | 1 (7%) |  |
|  |  |  |  |  |  |
| **Indigenous status** |  | |  |  | 0.62 |
| Indigenous | 26 (3%) | 16 (3%) | 10 (4%) | 0 (0%) |  |
| Not indigenous | 776 (97%) | 515 (97%) | 246 (96%) | 15 (100%) |  |
|  |  |  |  |  |  |
| **Country** |  |  |  |  | 0.25 |
| Australia | 647 (81%) | 425 (80%) | 212 (83%) | 10 (67%) |  |
| Other | 155 (19%) | 106 (20%) | 44 (17%) | 5 (33%) |  |
|  |  |  |  |  |  |
| **Fear of AI** [13] (mean, SD) | 6 (2) | 6 (2) | 6 (2) | 5 (2) | 0.53 |
|  |  |  |  |  |  |
| **Acceptance of AI** [13] (mean, SD) | 5 (2) | 5 (2) | 5 (2) | 6 (1) | 0.54 |
|  |  |  |  |  |  |
| **Risk attitudes**[14] |  |  |  |  | 0.064 |
| Not willing to take risks | 64 (8%) | 48 (9%) | 16 (6%) | 0 (0%) |  |
| 1 | 74 (9%) | 49 (9%) | 25 (10%) | 0 (0%) |  |
| 2 | 91 (11%) | 68 (13%) | 23 (9%) | 0 (0%) |  |
| 3 | 95 (12%) | 62 (12%) | 32 (13%) | 1 (7%) |  |
| 4 | 86 (11%) | 60 (11%) | 26 (10%) | 0 (0%) |  |
| 5 | 131 (16%) | 82 (15%) | 42 (16%) | 7 (47%) |  |
| 6 | 104 (13%) | 72 (14%) | 30 (12%) | 2 (13%) |  |
| 7 | 86 (11%) | 51 (10%) | 32 (13%) | 3 (20%) |  |
| 8 | 46 (6%) | 28 (5%) | 17 (7%) | 1 (7%) |  |
| 9 | 15 (2%) | 7 (1%) | 7 (3%) | 1 (7%) |  |
| Very willing to take risks | 10 (1%) | 4 (1%) | 6 (2%) | 0 (0%) |  |
|  |  |  |  |  |  |
| **Health Literacy** [15] |  | |  |  | <0.001 |
| Not at all | 7 (1%) | 3 (1%) | 4 (2%) | 0 (0%) |  |
| A little bit | 29 (4%) | 14 (3%) | 14 (5%) | 1 (7%) |  |
| Somewhat | 126 (16%) | 64 (12%) | 54 (21%) | 8 (53%) |  |
| Quite a bit | 247 (31%) | 163 (31%) | 80 (31%) | 4 (27%) |  |
| Extremely | 393 (49%) | 287 (54%) | 104 (41%) | 2 (13%) |  |
|  |  |  |  |  |  |
| **Education** |  | |  |  | 0.48 |
| Primary | 9 (1%) | 5 (1%) | 4 (2%) | 0 (0%) |  |
| Yr 10 | 145 (18%) | 93 (18%) | 50 (20%) | 2 (13%) |  |
| Yr 12 | 135 (17%) | 82 (15%) | 50 (20%) | 3 (20%) |  |
| TAFE | 310 (39%) | 217 (41%) | 88 (34%) | 5 (33%) |  |
| Undergrad | 155 (19%) | 98 (18%) | 54 (21%) | 3 (20%) |  |
| Postgrad | 48 (6%) | 36 (7%) | 10 (4%) | 2 (13%) |  |
|  |  |  |  |  |  |

**Supplementary Material References**

1. Voets MM, Veltman J, Slump CH, Siesling S, Koffijberg H. Systematic Review of Health Economic Evaluations Focused on Artificial Intelligence in Healthcare: The Tortoise and the Cheetah. Value in Health. 2022;25:340–9.

2. Wolff J, Pauling J, Keck A, Baumbach J. The Economic Impact of Artificial Intelligence in Health Care: Systematic Review. Journal of Medical Internet Research. 2020;22:e16866.

3. Soekhai V, de Bekker-Grob EW, Ellis AR, Vass CM. Discrete Choice Experiments in Health Economics: Past, Present and Future. PharmacoEconomics. 2019;37:201–26.

4. Mathioudakis AG, Salakari M, Pylkkanen L, Saz-Parkinson Z, Bramesfeld A, Deandrea S, et al. Systematic review on women’s values and preferences concerning breast cancer screening and diagnostic services. Psycho-Oncology. 2019;28:939–47.

5. Hendrix N, Hauber B, Lee CI, Bansal A, Veenstra DL. Artificial intelligence in breast cancer screening: primary care provider preferences. J Am Med Inform Assoc. 2020;28:1117–24.

6. Hendrix N, Lowry KP, Elmore JG, Lotter W, Sorensen G, Hsu W, et al. Radiologist Preferences for Artificial Intelligence-Based Decision Support During Screening Mammography Interpretation. Journal of the American College of Radiology. 2022;19:1098–110.

7. von Wedel P, Hagist C. Physicians’ preferences and willingness to pay for artificial intelligence-based assistance tools: a discrete choice experiment among german radiologists. BMC Health Services Research. 2022;22:398.

8. Nelson CA, Pérez-Chada LM, Creadore A, Li SJ, Lo K, Manjaly P, et al. Patient Perspectives on the Use of Artificial Intelligence for Skin Cancer Screening. JAMA Dermatol. 2020;156:1–12.

9. Ongena YP, Yakar D, Haan M, Kwee TC. Artificial Intelligence in Screening Mammography: A Population Survey of Women’s Preferences. J Am Coll Radiol. 2021;18:79–86.

10. de Vries CF, Morrissey BE, Duggan D, Staff RT, Lip G. Screening participants’ attitudes to the introduction of artificial intelligence in breast screening. J Med Screen. 2021;28:221–2.

11. Haan M, Ongena YP, Hommes S, Kwee TC, Yakar D. A Qualitative Study to Understand Patient Perspective on the Use of Artificial Intelligence in Radiology. Journal of the American College of Radiology. 2019;16:1416–9.

12. Lennox-Chhugani N, Chen Y, Pearson V, Trzcinski B, James J. Women’s attitudes to the use of AI image readers: a case study from a national breast screening programme. BMJ Health Care Inform. 2021;28:e100293.

13. Sindermann C, Sha P, Zhou M, Wernicke J, Schmitt HS, Li M, et al. Assessing the Attitude Towards Artificial Intelligence: Introduction of a Short Measure in German, Chinese, and English Language. Künstl Intell. 2021;35:109–18.

14. Dohmen T, Falk A, Huffman D, Sunde U, Schupp J, Wagner GG. Individual Risk Attitudes: Measurement, Determinants, and Behavioral Consequences. Journal of the European Economic Association. 2011;9:522–50.

15. Wallace LS, Rogers ES, Roskos SE, Holiday DB, Weiss BD. Brief Report: screening items to identify patients with limited health literacy skills. J GEN INTERN MED. 2006;21:874–7.
